# Vimentin expression as a prognostic marker in pancreatic cancer: a systematic review and meta-analysis

**DOI:** 10.3389/fmed.2026.1742644

**Published:** 2026-02-13

**Authors:** Oana-Iulia Cretu, Cristian Virgil Lungulescu, Manuel Gentiluomo, Adina Turcu-Stiolica, Nikola Panić, Stefan Patrascu, Bogdan Silviu Ungureanu

**Affiliations:** 1 Department of Pathology, Faculty of Medicine, University of Medicine and Pharmacy of Craiova, Craiova, Romania; 2 Department of Oncology, Faculty of Medicine, University of Medicine and Pharmacy of Craiova, Craiova, Romania; 3 Department of Biology, University of Pisa, Pisa, Italy; 4 Department of Biostatistics, Faculty of Pharmacy, University of Medicine and Pharmacy of Craiova, Craiova, Romania; 5 Department of Health Economics and Outcomes Research, Faculty of Medicine, University of Medicine and Pharmacy “Iuliu Haţieganu”, Cluj-Napoca, Romania; 6 Digestive Endoscopy Unit, University Hospital Center Dr. Dragiša Mišović Dedinje, Belgrade, Serbia; 7 Department of General Surgery, Faculty of Medicine, University of Medicine and Pharmacy of Craiova, Craiova, Romania; 8 Department of Gastroenterology, Faculty of Medicine, University of Medicine and Pharmacy of Craiova, Craiova, Romania

**Keywords:** meta-analysis, metastasis, overall survival, pancreatic cancer, vimentin

## Abstract

**Background:**

Vimentin, a key component of the epithelial-to-mesenchymal transition (EMT), has been mechanistically implicated in the progression and poor prognosis of pancreatic ductal adenocarcinoma (PDAC). However, clinical studies investigating the prognostic significance of tumor-cell vimentin expression have yielded inconsistent results. This systematic review and meta-analysis aimed to precisely quantify the association between vimentin expression and key clinicopathological features, including overall survival (OS), tumor stage, lymph node status, and distant metastasis.

**Methods:**

A systematic search was conducted across PubMed, Web of Science, and Scopus, published before July 8th, 2025, yielding 443 articles. Following duplicate removal and two rounds of independent screening using Rayyan by two reviewers, with non-concordance resolved by a third, a total of nine articles met the inclusion criteria for the meta-analysis. Pooled effect sizes were calculated using Hazard Ratios (HRs) for OS and Odds Ratios (ORs) with 95% Confidence Intervals (CIs) for categorical outcomes. Heterogeneity was assessed using the *I*^2^ statistic and *Q*-test, and publication bias was evaluated via funnel plot symmetry.

**Results:**

We demonstrated a statistically significant association between tumor-cell vimentin expression and reduced OS [pooled logHR = 1.39, 95% CI: (0.11, 2.68), *p* = 0.034], although high heterogeneity was observed (*I*^2^ = 93.64%, *p* < 0.001). Crucially, vimentin expression was also significantly associated with a higher lymph node stage [N-stage; pooled logOR = 0.58, 95% CI (0.08, 1.08), *p* = 0.022], with negligible heterogeneity (*I*^2^ = 0%, *p* = 0.418). In contrast, no significant association was found between vimentin expression and either primary T-stage [pooled logOR = −0.10, 95% CI: (−0.87, 0.66), *p* = 0.791] or M-stage [pooled OR = 0.08, 95% CI: (−0.95, 1.10), *p* = 0.882]. Publication bias was minimal for the N and T stages, but notable asymmetry was observed for the OS analysis.

**Conclusion:**

While tumor-cell vimentin expression is significantly associated with poorer OS and lymph node involvement, high heterogeneity and potential publication bias necessitate caution. Current evidence suggests vimentin is a promising prognostic indicator, but its clinical utility as a standalone biomarker remains limited by a lack of methodological standardization.

**Systematic Review Registration:**

PROSPERO 2025 CRD420251127404. Available from https://www.crd.york.ac.uk/PROSPERO/view/CRD420251127404.

## Introduction

Pancreatic cancer (PC) carries a heavy burden and a grim outlook due to its unfavorable prognosis, regardless of all available therapies (1). Outcomes remain poor, with no effective population to screen, and surgery as the only option to extend disease survival. However, only a minority of patients present with a resectable tumor (2). Median overall survival (OS) after pancreatectomy varies widely, with a 5-year survival being achievable but not guaranteed (3).

While pathology remains the gold standard for PDAC diagnosis, conventional grade and margin status only partially anticipate who will recur. Molecular subtyping can capture biological aggressiveness, but it is not yet uniformly available, standardized, or rapid enough to steer the daily practice (4). In both neoadjuvant and adjuvant settings, clinicians would benefit from biomarkers that may predict treatment response and early relapse, ideally ones that can be measured on endoscopic ultrasound guided biopsies, and that may be integrate with established clinicopathologic factors (5). The ideal marker would bridge diagnosis and prognosis, reflect tumor–stroma crosstalk, and help calibrate surveillance intensity and clinical-trial enrollment (6). As a practical read-out of epithelial to mesenchymal transition (EMT) and cellular plasticity, vimentin immunohistochemistry expression has the potential to fill this gap, potentially adding diagnostic prognostic stratification.

Vimentin is a type III intermediate filament and a canonical marker of mesenchymal differentiation. Its induction in pancreatic ductal adenocarcinoma (PDAC) reflects activation of the epithelial–mesenchymal transition (EMT) process (7). This creates an important interpretation issue: vimentin staining is often strong in the desmoplastic stroma, fibroblasts/CAFs and other mesenchymal components as expected for mesenchymal lineage, while a subset of tumor epithelial cells can also show aberrant/EMT-associated vimentin positivity (8). Moreover, it has been suggested that it might be a central regulator that enables cancer cell survival and migration during progression. This may be linked to its mechanical properties as they may assemble a viscoelastic network that compresses forces during invasion and creates a perinuclear cover that helps preserve the nuclear envelope integrity (9). Thus, a matrix-degrading protrusion may occur, which may be linked to the dissemination process in PDAC (10).

Prior clinical-pathology studies support prognostic relevance for both compartments: epithelial vimentin expression in pancreatic adenocarcinoma has been associated with worse post-surgical survival (including a commonly used >10% positive-tumor-cell cutoff in one cohort) (4), and in resected PDAC, tumor vimentin by immunohistochemistry has been reported as an adverse prognostic factor alongside other proliferative/stemness markers (11). Complementing this, stromal vimentin, particularly in specific CAF subpopulations, has also been linked to shorter overall survival in PDAC (12), suggesting that vimentin can carry prognostic information whether it marks EMT-like tumor cells or distinct, outcome-relevant stromal biology. While vimentin is not diagnostic for PDAC, integrating its tumor-cell expression with budding and subtype markers refines risk stratification beyond standard clinicopathologic variables (11).

To address whether tumor-cell vimentin expression in PDAC may be relevant for prognosis assessment, we conducted a meta-analysis.

## Materials and methods

### Literature searching

Our research was based on the Preferred Reported Items for Systematic Reviews and Meta-analyses (PRISMA) checklist (Table S1 in Supplementary material S1) (13). The relevant literatures were searched from Web of Science, Pubmed, and Scopus. We retrieved articles using the following Boolean search terms (“vimentin”) AND (“pancreatic cancer” OR “pancreatic ductal adenocarcinoma”) AND (“immunohistochemistry”; as shown in Table S2 in Supplementary material S2). The literature searching was available until July 8th, 2025. No language restrictions were applied at the search level to avoid “language bias.”

Inclusion criteria: (1) histologically confirmed PDACPDAC; (2) case-control or cohort studies; (3) assessment of vimentin in primary tumor cells; (4) reported prognostic or clinicopathological data.

Exclusion criteria: (1) animal studies (murine/porcine); (2) *in vitro* studies; (3) systematic-reviews or meta-analysis; (4) studies that data was insufficient to calculate OR or HR value; (5) case reports, editorials, commentaries, and conference abstracts (unless they provided sufficient quantitative data for HR/OR extraction).

### Data extraction, quality assessment, and publication bias

Two authors (I-O.C. and B.S.U.) screened titles, abstracts, and full texts for eligibility, using Ryann (14), a web-based tool designed for systematic reviews and meta-analyses, and extracted data from the included studies independently. The process involved: blinded screening (each reviewer independently categorized records as “Include,” “Exclude,” or “Maybe,” with conflicts automatically flagged by Rayyan), and full-text review (screened abstracts were advanced to full-text assessment if marked “Include” or “Maybe” by either reviewer). Extracted data included study characteristics, population demographics, methods of vimentin assessment, and clinical features (tumor stage, lymph node metastasis, histological differentiation, stage, lymphatic invasion, venous invasion, neural invasion). Data on vimentin immunoexpression were extracted as originally reported, scoring was limited to tumor cells when specified, and studies without clear differentiation between tumor and stromal staining were included as such, this methodological variability being recognized as a limitation. Disagreements were resolved by consensus or a third reviewer (A.T-S.). The quality of included studies was assessed using the Newcastle-Ottawa Scale (NOS) for cohort studies (15).

Publication bias was assessed via visual inspection of funnel plots and, where applicable, by Kendall's Tau correlation and Egger's regression test to check for funnel plot asymmetry.

### Statistical analysis

R (version 4.5.1–1.2204.0, R Foundation, Vienna, Austria) with the metafor package (16) and RevMan (version 5.4, Cochrane Collaboration, 2020) were used. Hazard ratios (HRs) with 95% CIs were used to assess the association between OS of PC and vimentin expression. Pooled effect sizes were calculated using Odds Ratios (ORs) with 95% Confidence Intervals (CIs) for categorical outcomes. In instances where HRs were not directly reported, survival data were extracted from Kaplan-Meier curves using WebPlot Digitizer (Version 4.7, Austin, Texas, USA) (17). Meanwhile, logOR or logHR with 95% CIs was applied to observe the correlations clinical features of PC with vimentin expression. Statistical pooling was performed using either fixed-effects or random-effects models. The choice between models was guided by the presence of heterogeneity (*I*^2^ and Cochran's *Q* test). Where heterogeneity was low (*I*^2^ = 0% or *p* > 0.10), a fixed-effects model using the inverse-variance method was applied. In cases of significant heterogeneity (*I*^2^ > 0%), a random-effects model was utilized with the Restricted Maximum Likelihood (REML) estimator. REML was chosen over the traditional DerSimonian–Laird approach to provide a more robust estimate of between-study variance (τ^2^) given the number of studies included. Given the methodological heterogeneity between studies, including differences in immunohistochemical protocols, scoring systems, and cut-off for defining vimentin expression, the classification of “low/high” was performed according to the original definitions of each study. Pooling was performed using these specific classifications to preserve internal validity and avoid reclassification of cases by applying uniform cut-off in a heterogeneous methodological context. The amount of heterogeneity was reported with tau^2^, *Q*-test for heterogeneity results and the *I*^2^ statistic. In case of any amount of heterogeneity was detected (tau^2^ > 0, regardless of the results of the *Q*-test), a prediction interval for the true outcomes was also provided. Studentized residuals and Cook's distances were used to examine whether studies may be outliers and/or influential in the context of the model. Studies with a Cook's distance larger than the median plus 6 times the interquartile range of the Cook's distances are considered to be influential.

## Results

### Electronic search results and study characteristics

**Figure 1:**
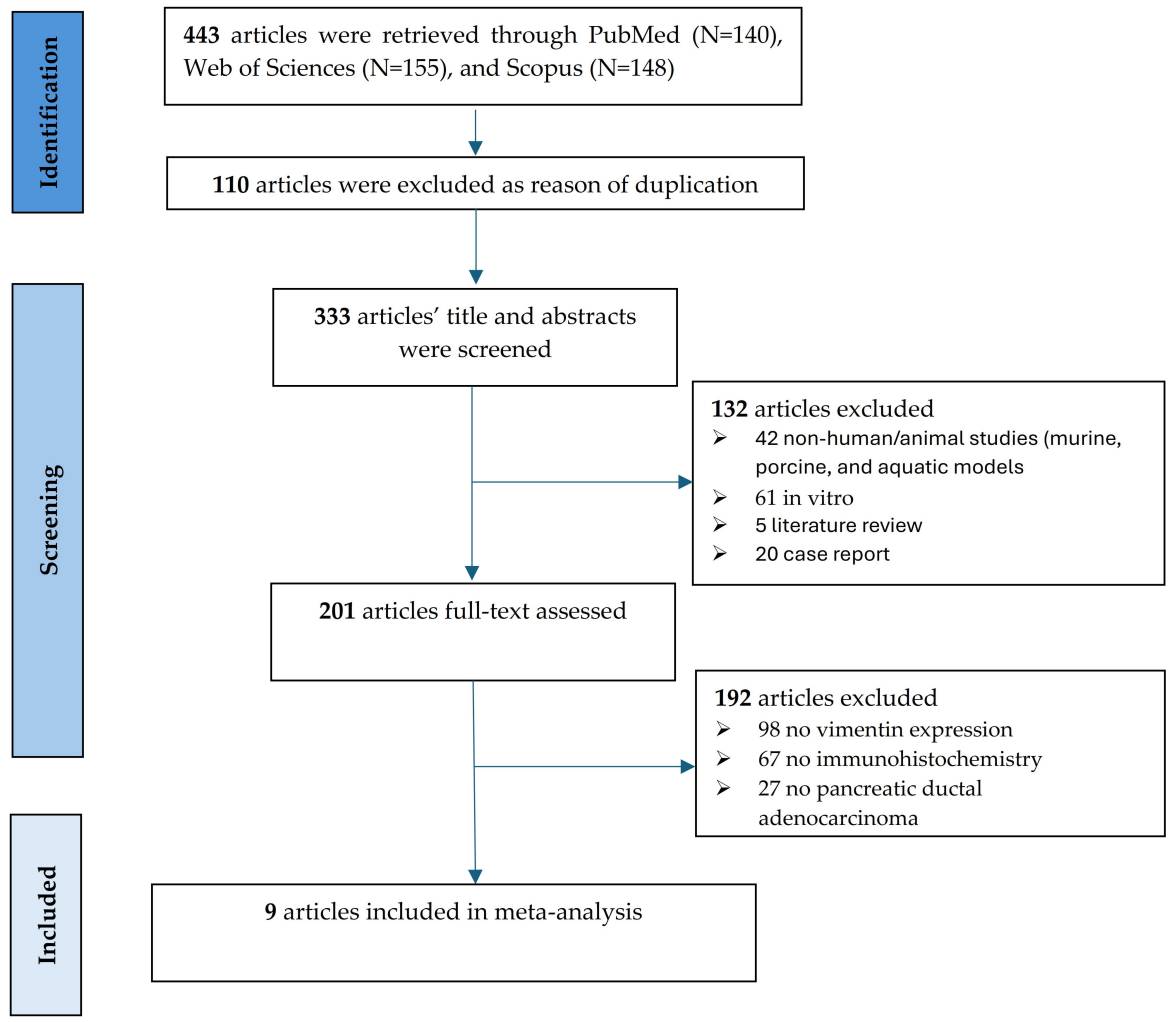
PRISMA flow chart of the literature search and study selection process.

A PRISMA flow diagram (Figure 1) was used to document the study selection process for this systematic review and meta-analysis of vimentin in pancreatic cancer. The initial search across three databases: PubMed (*N* = 140), Web of Sciences (*N* = 155), and Scopus (*N* = 148), yielded a total of 443 articles. In the Identification phase, 110 articles were excluded due to duplication, leaving 333 articles for title and abstract screening. This initial screening was performed independently by two reviewers (O-I.C. and B.S.U.) utilizing the Rayyan systematic review software. During the Screening phase, 132 articles were excluded for various reasons, including: 42 non-human *in vivo* studies (comprising various animal models such as murine, porcine, and aquatic) and 61 *in vitro* cell-line studies; five literature reviews; and 20 case reports. This resulted in 201 articles undergoing full-text assessment, which was also conducted independently by the two reviewers. Any discrepancies regarding inclusion were resolved through consensus or by arbitration with a third reviewer (A.T-S.). Of these, 192 articles were excluded, primarily because they lacked vimentin expression data (*N* = 98), did not use immunohistochemistry (*N* = 67), or did not focus on pancreatic ductal adenocarcinoma (*N* = 27). Ultimately, a consensus was reached, and nine articles were selected and included in the final quantitative synthesis (meta-analysis), containing 721 PC patients, 558 low expression vimentin and 163 with high expression vimentin. Table 1 contains the characteristics of the included studies (18–26). Stage classification was evaluated using UICC 7th TNM classification of the Union for International Cancer Control in one study (22, 24) or the 6th edition AJCC in two studies (19, 25).

**Table 1:** Characteristics of the studies included in the meta-analysis.

**Study**	**Country**	**Patients number (Low/High)**	**Median age median (range)/mean ±SD**	**Cutoff/*H*-score**	**Method of the assessment of vimentin IHC expression**			**NOS**
					**Clone**	**Dilution**	**Retrieval**	
Chouat 2017 (19)	Tunisia	50 (28/22)	57 (29-78)	H-scores ≥5%	Mouse monoclonal anti-vimentin antibody, VIM-572, SRL33, Novocastra, Leica	1:400	Citrate buffer (HIER), pH 6.0	9
Guo 2014 (20)	China	76 (49/27)	65 (40–81)	*H*-scores ≥5%	Mouse monoclonal anti-vimentin antibody, Santa Cruz	1:100	Citrate buffer (HIER), pH 6.0	4
Javle 2007 (21)	USA	34 (30/4)	66 (38–84)	*H*-scores >100	Mouse monoclonal anti-vimentin antibody Vim3B4, Dako, CA	–	Citrate buffer (HIER), pH 6.0	7
Kokumai 2023 (22)	Japan	120 (90/30)	67 (41–89)	*H*-scores ≥10%	Mouse monoclonal anti-vimentin antibody, V9, Santa Cruz Biotechnology	1:100	Citrate buffer (HIER), pH 6.0	8
Lee 2014 (23)	South Corea	64 (54/10)	65.3 ± 9.0	*H*-scores ≥2	Mouse monoclonal anti-vimentin antibody, DAKO	1:1,200	Citrate buffer (HIER), pH 6.0	8
Maehira 2019 (24)	Japan	67 (43/24)	71 (62–76)	*H*-scores ≥5%	Rabbit monoclonal anti-vimentin antibody, #5741S, Cell Signaling Technology, Inc., Danvers, MA	1:100	Citrate buffer (HIER), pH 6.0	9
Sanchez Ramirez 2022 (25)	USA	130 (106/24)	63 (41–82)	*H*-scores ≥1	Mouse recombinant monoclonal anti-vimentin, NSJ Bioreagents, San Diego, CA, USA	1:1,000	Citrate buffer (HIER), pH 6.0	9
Wang 2019 (18)	USA	120 (106/14)	63.9 (42–84)	*H*-scores ≥10%	Mouse monoclonal anti-vimentin antibody, clone V9, Dako, Carpinteria, Calif.	1:900	Citrate buffer (HIER), pH 6.0	8
Xu 2013 (26)	China	60 (52/8)	61.7 ± 12.41	*H*-scores ≥20%	Mouse monoclonal anti-vimentin antibody, clone V9, Dako	1:100	Citrate buffer (HIER), pH 6.0	7

IHC, immunohistochemical; HIER, Heat-Induced Epitope Retrieval.

The methodological quality of the studies was assessed using the Newcastle-Ottawa Scale (NOS), with scores ranging from 4 to 9, as shown in Table S3 of Supplementary material S2. The highest quality was observed in the studies conducted by Chouat et al. (19), Maehira et al. (24) and Sánchez-Ramírez et al. (25), each achieving 9/9 points through rigorous cohort selection, appropriate multivariable adjustment and complete follow-up reporting. The studies by Kokumai et al. (22), Lee et al. (23) and Wang et al. (18) scored 8/9. These studies included well-defined cohorts, applied appropriate exposure assessment methods and performed adjustments for relevant confounders. However, they did not clearly report the proportion of patients lost to follow-up or provide an explicit statement regarding the percentage of patients followed until the endpoint. Consequently, although the internal validity of the association appears strong, the uncertainty regarding follow-up losses requires caution when interpreting the risk of bias related to follow-up. Two studies reached only 7/9 points. The study by Xu et al. (26) lost points in the comparability domain due to insufficient details on multivariable adjustment for key confounders, as well as in the adequacy of follow-up, since the proportion of patients lost was not reported, while Javle et al. (21) applied a limited multivariable model and provided insufficient follow-up data, raising concerns about potential bias. Guo et al. (20) obtained the lowest score (4/9) reflecting modest methodological quality. The outcome of interest was assessed at the time of resection rather than as a subsequent event, no multivariable adjustments were performed, and analyses were restricted to simple statistical tests. The authors explicitly acknowledged these limitations and did not provide survival data.

### Correlations between vimentin and T stage (T3–T4 vs. T1–T2)

**Figure 2:**
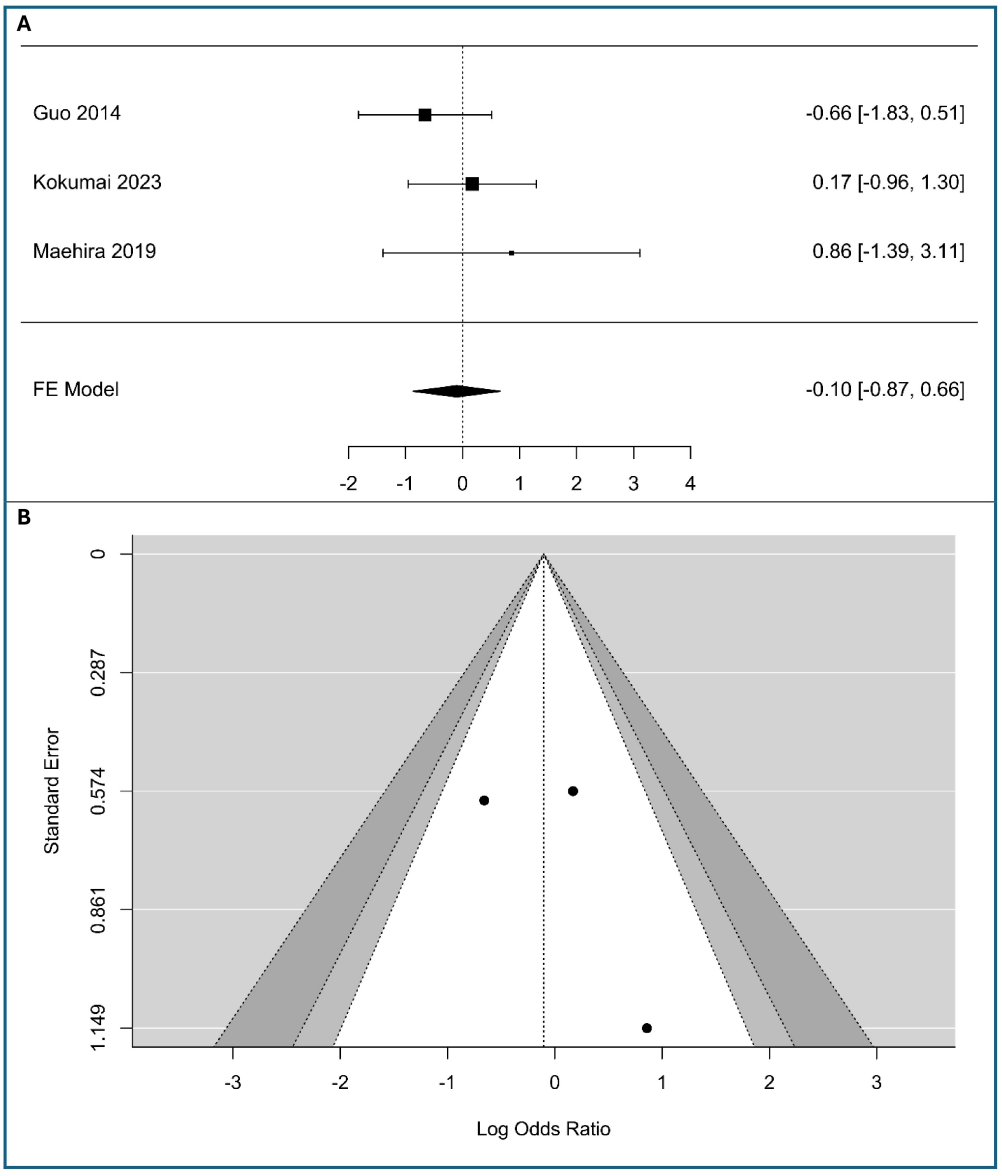
Correlation between tumor-cell vimentin expression and T-stage in Pancreatic Ductal Adenocarcinoma. **(A)** Forest plot showing the pooled log Odds Ratios for T-stage (T3-T4 vs. T1-T2). **(B)** Funnel plot for the assessment of publication bias.

A meta-analysis was performed on three studies (*n* = 263 patients) to investigate the association between vimentin expression and tumor stage in PC, including 192 patients with low expression vimentin and 71 patients with high expression vimentin, as shown in Figure 2A. The observed logOR ranged from −0.658 to 0.858, with the majority of estimated being positive (67%). The estimated average logOR based on fixed-effects model was −0.10 (95% CI: −0.87 to 0.66). Therefore, the average outcome did not differ significantly from zero (*z* = −0.27, *p* = 0.791). Based on the absence of significant heterogeneity as determined by the *Q*-test (*Q*(2) = 1.79, *p* = 0.41, *I*^2^ = 0%), a fixed-effects model was employed. This confidence interval crosses the line of no effect (0), indicating that there is no statistically significant association between vimentin expression and T-stage in this meta-analysis. Clinically, these data suggest that while vimentin is a marker of cellular migration, its expression does not correlate with primary tumor size or local invasiveness (T-stage) in PDAC, indicating it may be a more specific indicator of metastatic spread rather than local tumor growth.

Neither the rank correlation nor the regression test indicated any funnel plot asymmetry (*p* = 1.0 and *p* = 0.41, respectively). The funnel plot in Figure 2B appears symmetrical, with studies distributed relatively evenly around the pooled effect. The studies, despite their varying precision, fall within the expected funnel shape. This symmetry suggests that there is no apparent publication bias in the included literature regarding the association between vimentin expression and T-stage. The meta-analysis results are likely robust against bias, though the small number of studies means caution is warranted in overinterpreting symmetry.

### Correlations between vimentin and stage N (N1 vs. N0)

**Figure 3:**
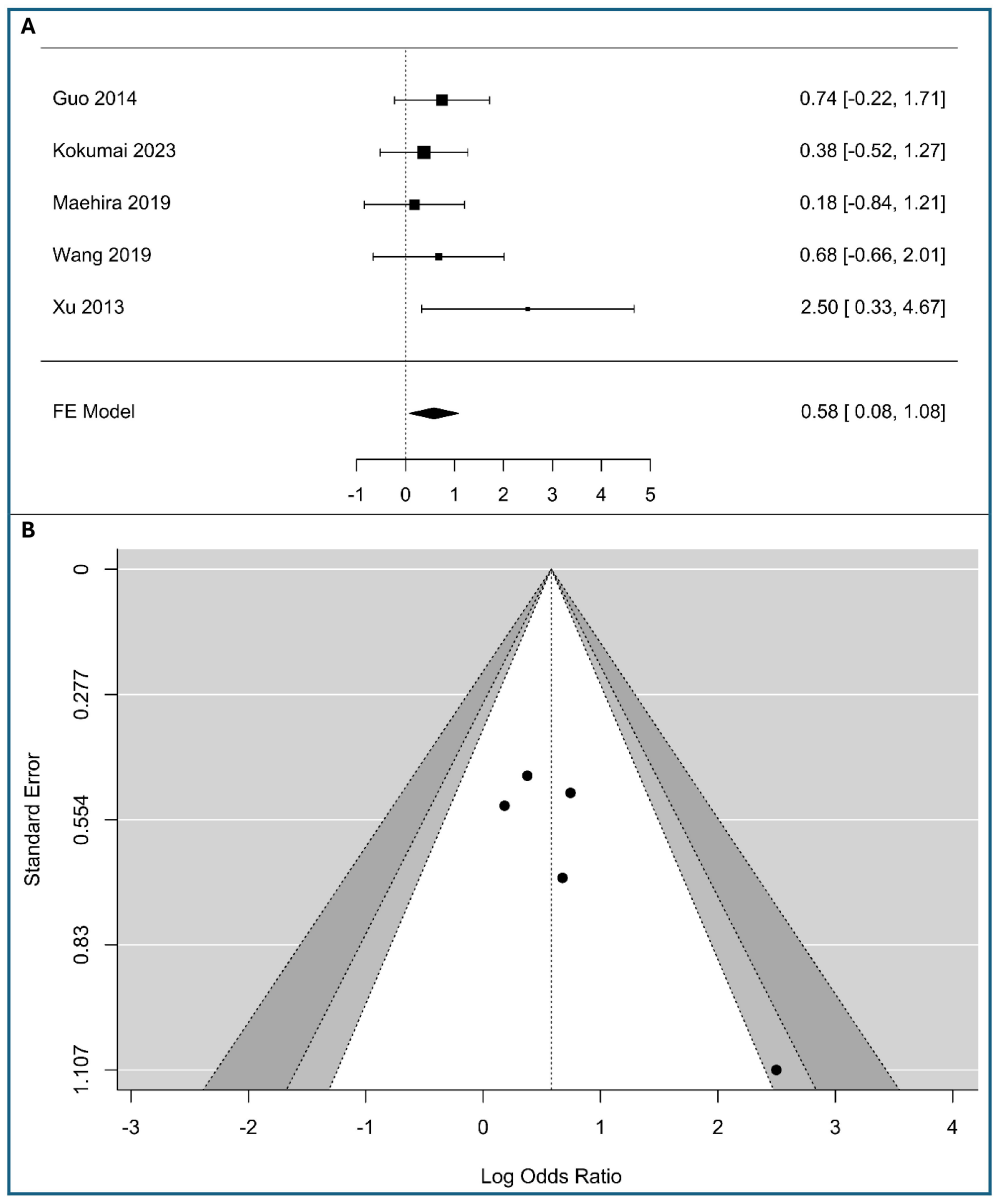
Correlation between tumor-cell vimentin expression and N-stage in Pancreatic Ductal Adenocarcinoma. **(A)** Forest plot showing the pooled log Odds Ratios for N-stage (N1 vs. N0). **(B)** Funnel plot for the assessment of publication bias.

A meta-analysis of five studies was conducted to assess the association between tumor-cell vimentin expression and lymph node metastasis, including in the analysis (340 patients with low expression vimentin and 103 patients with high expression vimentin), as shown in Figure 3A. The observed logOR ranged from 0.182 to 2.498, with all of the estimated being positive. The estimated average logOR based on the fixed-effects model was 0.58 (95% CI: 0.083–1.078), with the average outcome significantly different from zero (*z* = 2.29, *p* = 0.022), indicating a statistically significant positive association between vimentin expression and the presence of lymph node metastasis. The significant association with nodal involvement suggests vimentin could help identify patients at high risk for occult lymphatic metastasis who might benefit from more extensive lymphadenectomy.

The forest plot visually confirms this result. While the individual studies—Guo et al. (20), Kokumai et al. (22), Maehira et al. (24), and Wang et al. (11)—show wide confidence intervals that cross the line of no effect, the study by Xu et al. (26) demonstrates a significant positive association [2.50, 95% CI: (0.33, 4.67)]. The overall pooled result is driven by the consistent, albeit non-significant, trend toward a positive association across most studies, culminating in a statistically significant combined effect. The minimal heterogeneity (*I*^2^ = 0%) suggests that the true effect is consistent across the included studies.

According to the *Q*-test, there was no significant amount of heterogeneity in the true outcomes (*Q*(4) = 3.91, *p* = 0.42, *I*^2^ = 0%). The funnel plot does not indicate significant asymmetry, as shown in Figure 3B and Table S4 of Supplementary material S2, supporting the robustness of the meta-analysis results, assuming no other biases are present.

### Correlations between vimentin and metastasis (M1 vs. M0)

**Figure 4:**
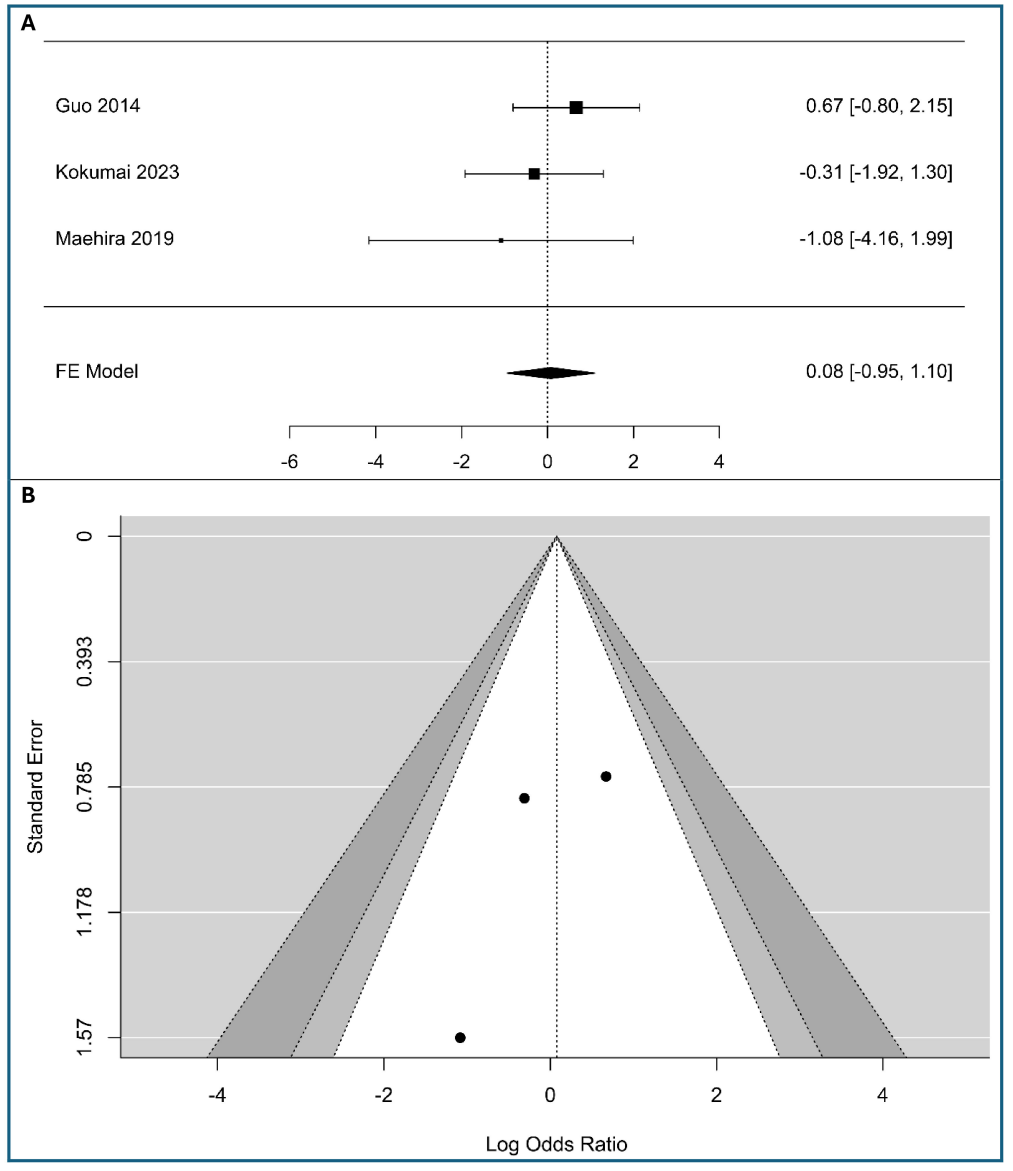
Correlation between tumor-cell vimentin expression and Metastasis in Pancreatic Ductal Adenocarcinoma. **(A)** Forest plot showing the pooled log Odds Ratios for N-stage (M1 vs. M0). **(B)** Funnel plot for the assessment of publication bias.

A meta-analysis was conducted on three studies to examine the association between tumor-cell vimentin expression and metastasis in PC, including 182 patients with low expression vimentin and 81 patients with high expression vimentin, as shown in Figure 4A. The observed logOR ranged from −1.082 to 0.67, with the majority of estimated being negative (67%). The estimated average logOR based on fixed-effects model was 0.08 (95%CI: −0.95 to 1.10), with average outcome different significantly from zero (*z* = 0.15, *p* = 0.88). The confidence interval for the pooled effect size crosses the line of no effect (0), indicating that the association between vimentin expression and metastasis is not statistically significant in this analysis. The confidence intervals for all three studies are wide and cross the line of no effect, meaning that none of the individual studies demonstrated a statistically significant association. The lack of association with distant metastasis suggests that vimentin-driven EMT may be a more localized driver of lymphatic spread than of systemic hematogenous dissemination.

According to the *Q*-test, there was no significant amount of heterogeneity in the true outcomes (*Q*(2) = 1.39, *p* = 0.498, *I*^2^ = 0%). The visual inspection of the forest plot suggests also low heterogeneity, as the confidence intervals of the individual studies largely overlap. The funnel plot (Figure 4B) and Table S3 in Supplementary material S2 show no asymmetry, indicating minimal risk of publication bias. While results are robust, the limited study count advises cautious interpretation.

### Correlations between vimentin and histological stage (III+IV vs. I+II)

**Figure 5:**
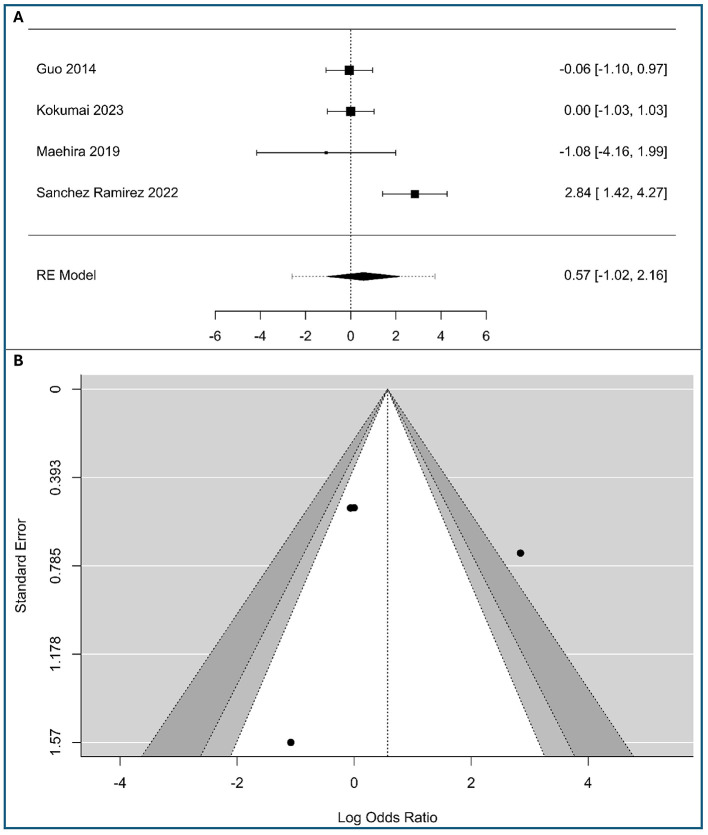
Correlation between tumor-cell vimentin expression and Histological stage in Pancreatic Ductal Adenocarcinoma. **(A)** Forest plot showing the pooled log Odds Ratios for Histological stage (III+IV vs. I+II). **(B)** Funnel plot for the assessment of publication bias.

A total of four studies were included in the analysis (298 patients with low expression vimentin and 105 patients with high expression vimentin), as shown in Figure 5A. The observed logOR ranged from −1.082 to 2.84, with half of estimated being negative (50%). The estimated average logOR based on random-effects model was 0.57 (95% CI: −1.02 to 2.16), with average outcome did not differ significantly from zero (*z* = 0.71, *p* = 0.48). According to the *Q*-test, there was significant amount of heterogeneity in the true outcomes (*Q*(3) = 13.39, *p* = 0.0039, τ^2^ = 1.95, *I*^2^ = 80.56%), likely due to the use of different staging systems (UICC 7th vs. AJCC 6th). A 95% prediction interval for the true outcomes is given by −2.59 to 3.74. Hence, although the average outcome is estimated to be positive, in some studies the true outcome may be negative. An examination of the studentized residuals revealed that one study (25) had a value larger than ± 2.394 and may be a potential outlier in the context of this model. According to the rank correlation, the regression test (Table S3 in Supplementary material S2) and the funnel plot asymmetry (Figure 5B), minimal risk of publication bias was observed. While results are robust, the limited study count advises cautious interpretation.

Clinically, these results suggest that vimentin-driven mesenchymal transition is not a reliable predictor of the overall TNM stage grouping, indicating that vimentin expression may occur independently of the cumulative anatomic extent of the disease in PDAC.

### Correlations between vimentin and histological grade (poorly vs. well/moderate differentiated)

**Figure 6:**
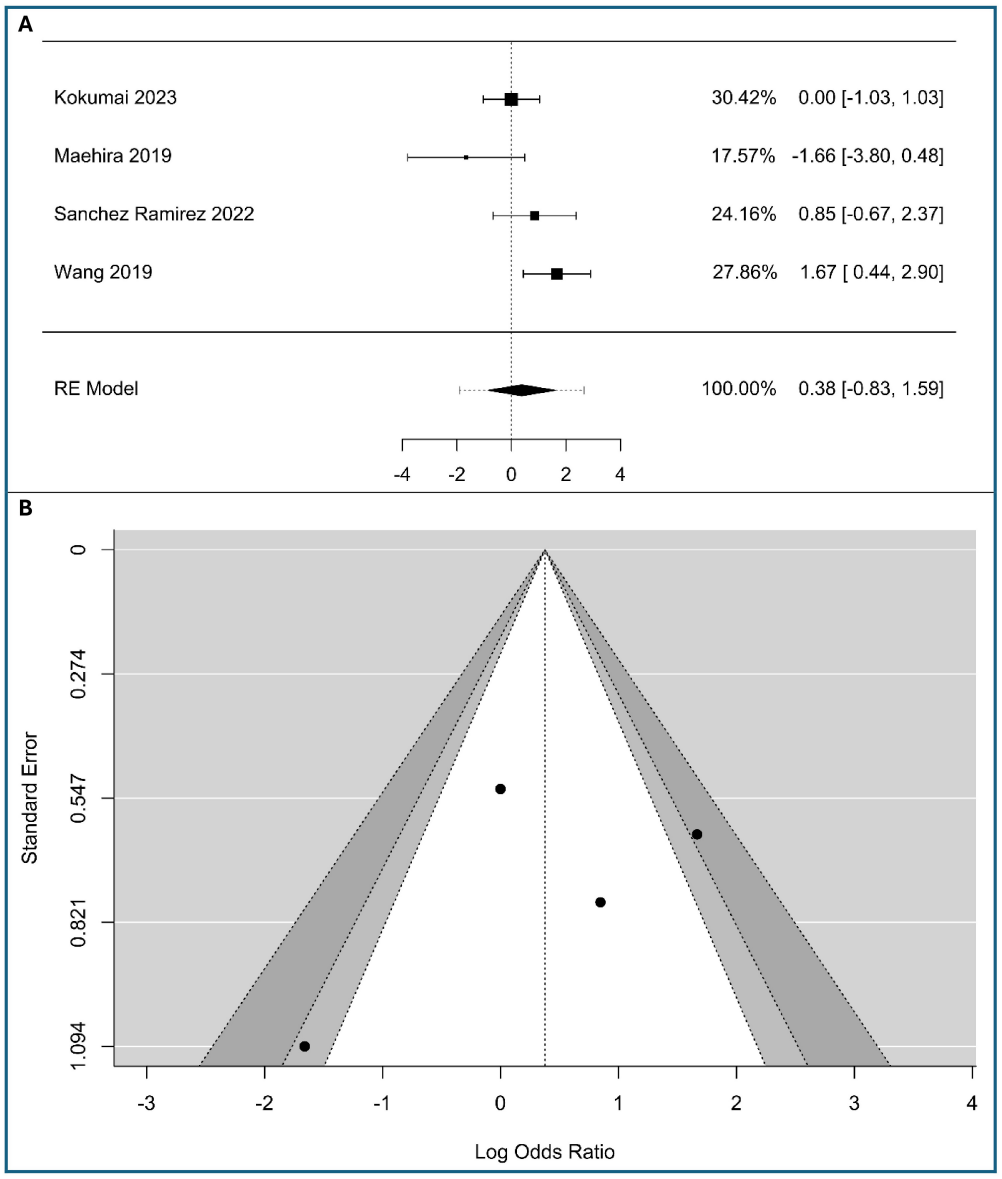
Correlation between tumor-cell vimentin expression and Histological grade in Pancreatic Ductal Adenocarcinoma. **(A)** Forest plot showing the pooled log Odds Ratios for Histological grade (poorly vs. well/moderate differentiated). **(B)** Funnel plot for the assessment of publication bias.

A total of three studies were included in the analysis, as shown in Figure 6A. The observed logOR ranged from −1.66 to 1.67, with the majority of estimated being negative (67%). The estimated average logOR based on random-effects model was 0.46 (95%CI: −1.37 to 2.28), with average outcome did not differ significantly from zero (*z* = 0.49, *p* = 0.624). According to the *Q*-test, there was no significant amount of heterogeneity in the true outcomes (*Q*(2) = 6.96, *p* = 0.031, τ^2^ = 1.912, *I*^2^ = 74.57%). From a clinical perspective, this implies that the EMT marked by vimentin is a distinct biological phenomenon that can occur across the spectrum of histological differentiation, rather than being confined to poorly differentiated tumors. A 95% prediction interval for the true outcomes is given by −2.811 to 3.72. Hence, although the average outcome is estimated to be positive, in some studies the true outcome may be negative. The Egger's regression test (Table S3 in Supplementary material S2) indicated funnel plot asymmetry (*p* = 0.0087) but not the Begg's rank correlation test, which is more conservative and less powered with very few studies, like in our case (*p* = 0.333). The visual asymmetry (Figure 6B) and Egger's significant result suggest a potential small-study effect or publication bias, but this is a tentative conclusion, given the small number of studies.

### Association of vimentin and OS

**Figure 7:**
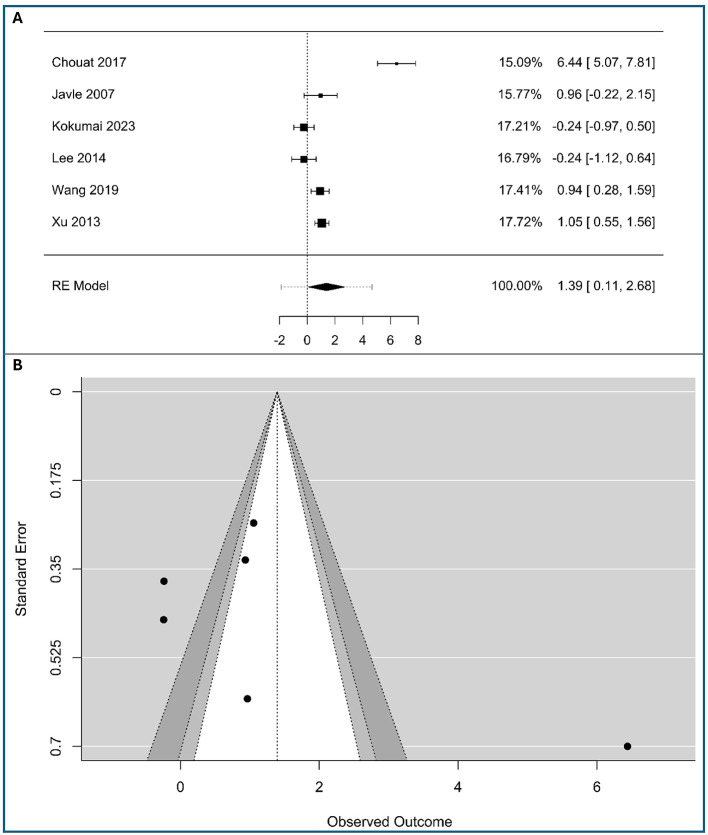
Association between tumor-cell vimentin expression and Overall Survival in Pancreatic Ductal Adenocarcinoma. **(A)** Forest plot showing the pooled log hazard ratios for overall survival. **(B)** Funnel plot for the assessment of publication bias.

A meta-analysis of six studies, including 360 patients with low vimentin expression and 84 patients with high vimentin expression, evaluated the impact of high vimentin expression on overall survival in pancreatic cancer, as shown in Figure 7A. The pooled analysis showed that high vimentin expression was significantly associated with worse OS, with a combined HR of 1.39 (95% CI: 0.11–2.68), based on a random-effects model, as shown in Figure 7A. However, there was substantial heterogeneity among studies (*Q*(5) = 78.68, τ^2^ = 2.36, *I*^2^ = 93.64%, *p* < 0.001), suggesting considerable variation in effect sizes. The overall effect estimate remained statistically significant despite this heterogeneity, as shown by the *Z* statistic (*Z* = 2.12, *p* = 0.034), suggesting that high vimentin expression may be a negative prognostic factor for survival in pancreatic cancer. The increased hazard ratio for overall survival indicates that tumor-cell vimentin status serves as a potent indicator of poor biological behavior and shorter life expectancy in PDAC.

The high heterogeneity is visually apparent in the forest plot, as the individual study confidence intervals do not overlap significantly. For example, the study by Chouat et al. (19) shows a large positive effect (6.44), while the studies by Kokumai et al. (22) and Lee et al. (23) show a negative effect (−0.24). The remaining studies show varying degrees of positive effect. This wide range of outcomes highlights the importance of using a random-effects model, which acknowledges and incorporates this variability into the final pooled estimate. The weighting of each study, as shown in the forest plot, indicates their proportional contribution to the overall effect.

Visual inspection of the funnel plot (Figure 7B) suggested mild asymmetry, with most of the studies on the left side of the funnel (a potential for publication bias), possibly indicating small-study effects. However, Egger's regression test was statistically significant (*p* = 0.026), suggesting that there is strong evidence of publication bias, as in Table S3 in Supplementary material S2.

## Discussion

Our meta-analysis suggests that tumor-cell vimentin positivity associates with inferior OS in PC [pooled random-effects HR = 1.39, 95% CI of (0.11–2.68), *p* = 0.034], even though with marked heterogeneity (*Q*(5) = 78.68, *I*^2^ = 94%, *p* < 0.001). Clinically, this pooled HR of 1.39 supports the concept that EMT-leaning tumors are harder to eradicate systemically and more likely to drive mortality despite locoregional control. This underscores that vimentin is not just a passenger of the EMT process, but a significant independent indicator of poor patient outcome in PDAC. Given the heterogeneity, vimentin should not be used alone to guide therapy, but may be considered for the risk-stratification process.

Vimentin is widely expressed in stromal elements, so stromal staining should function only as an internal control (27). Quantification should be restricted to tumor cells confirmed by co-expression of cytokeratin. Variability in antibody clone, epitope retrieval, scoring thresholds (percent positive vs. *H*-score), and the anatomic area sampled (invasive front vs. tumor core) can materially affect results and likely contribute to the observed heterogeneity in survival associations (28). In addition, post-treatment specimens commonly display focal increases in vimentin with concomitant loss of membranous E-cadherin, consistent with therapy-induced EMT (7). While we acknowledge vimentin's role in the EMT panel, the high heterogeneity (*I*^2^ = 94%) observed in our OS analysis highlights the urgent need for a standardized “tumor-cell-only” IHC rubric specifically for vimentin. This variability suggests that the prognostic weight of vimentin is highly sensitive to scoring thresholds, and its future clinical utility depends on establishing vimentin-specific protocols rather than generic EMT assessments.

Our meta-analysis reveals that high tumor-cell vimentin expression is associated with a significantly greater likelihood of lymph-node metastasis at presentation [pooled effect size = 0.58, 95% CI = (0.08, 1.08), *p* = 0.022]. The significant pooled effect size, combined with the lack of heterogeneity among studies, strengthens the conclusion that vimentin expression is a reliable indicator of a more advanced nodal stage. The significant association with nodal involvement (OR = 0.58) found in our analysis provides direct evidence that vimentin-positive tumor cells possess a heightened capacity for lymphatic invasion. Unlike general EMT markers, our results specifically pinpoint vimentin expression as a reliable red flag for advanced nodal stage (N1), suggesting it could serve as a specific “metastatic switch” that helps clinicians anticipate lymphatic spread before surgery. This is plausible since vimentin upregulation marks activation of the EMT and cytoskeletal remodeling that promotes motility, invasion, and intravasation. Thus, this process is expected to facilitate transit into lymphatic channels and early nodal seeding. Moreover, it may be useful to refine pre-operative risk stratification after endoscopic ultrasound fine needle biopsy and may aid in counseling patients and prioritizing closer nodal assessment and surveillance (29).

We examined whether tumor-cell vimentin expression is associated with two baseline pathologic features in PDAC: advanced histological stage (III–IV vs. I–II) and poor differentiation. Taken together, these results suggest that vimentin positivity in tumor cells is not consistently associated with either a more advanced stage or poorer differentiation at diagnosis. The combination of substantial heterogeneity, wide confidence/prediction intervals, and potential outliers suggests caution in drawing firm. Importantly, the absence of a robust cross-sectional association with stage or grade does not negate the prognostic relevance of vimentin observed in individual cohorts. However, it highlights that EMT-related biology may transcend conventional morphological categories. Clinically, these findings suggest that vimentin should not be relied upon as a stand-alone surrogate for stage or grade at diagnosis. Instead, if vimentin is to be used for risk stratification, it should be interpreted in a panel (e.g., with E-cadherin loss, ZEB1, tumor budding) and in context (site within tumor, treatment exposure, molecular subtype) (17, 30, 31). From a translational perspective, this aligns with the concept that EMT/vimentin reflects plasticity and migratory competence that may be present early and evolve independently of baseline grade, making it more informative for recurrence risk or treatment response than for initial anatomic stage (11).

In the three available studies that allowed the study of T1+T2 vs. T3+T4, tumor-cell vimentin showed no statistically significant association with the baseline parameter under evaluation (pooled fixed-effects logOR −0.335; 95% CI −1.373 to 0.703), with low heterogeneity and no influential outliers. These findings are inconclusive rather than negative, given the wide confidence bounds and the small number of included studies. Our finding that vimentin does not correlate with T-stage or Histological Grade is a critical observation that defines vimentin's specific role. It demonstrates that tumor-cell vimentin marks a functional change in cell motility rather than a simple increase in cell proliferation or tumor mass. This indicates that vimentin adds value specifically by identifying “biologically advanced” tumors that might otherwise appear “anatomically early” (low T-stage) on standard imaging or pathology. Clinically, this suggests that vimentin should not be used as a stand-alone surrogate for that specific baseline feature at the time of diagnosis. In a similar manner for the metastasis status, no statistically significant association was found.

The results of our meta-analysis support vimentin as a promising marker of the EMT for overall survival and lymph node involvement. However, prospective studies with pathology standardization and clinically relevant cut-offs need to be implemented to provide a more accurate perspective. Also, vimentin should be considered in a multimarker EMT panel to provide a more rigorous prognostic value.

The study has limitations. A notable limitation is the potential for publication bias in the studies included in the OS meta-analysis, as the absence of studies with null or negative findings could inflate the observed effect size and lead to an overly optimistic conclusion about the strength of the association. Future work should prioritize standardization and granularity. Prospective cohorts or individual-patient-data meta-analyses with prespecified IHC protocols (clone, scoring rubric, tumor-cell–only assessment), *a priori* cut-offs (e.g., >5%, >10% and *H*-score thresholds), and stratification by specimen type (biopsy vs. resection), anatomic region (invasive front vs. bulk), and treatment status (treatment-naïve vs. post-neoadjuvant) are needed. Despite these limitations, the current findings provide a compelling case for the clinical relevance of vimentin as a prognostic biomarker in PDAC.

## Conclusions

Our meta-analysis suggests high vimentin expression was significantly associated with worse OS and with a greater likelihood of lymph-node metastasis at presentation, with potential clinical implications for staging and treatment planning. These findings may support the notion that vimentin has prognostic relevance and could be considered for risk stratification of patients. However, from a clinical perspective, caution is warranted, as it should be considered in conjunction with other EMT features. Standardizing IHC protocols—specifically regarding antibody selection and clinically validated cut-off values—is essential to transition vimentin from a research tool to a reliable clinical biomarker for pancreatic cancer prognosis.

## Data Availability

The raw data supporting the conclusions of this article will be made available by the authors, without undue reservation.
