# Correction: Vimentin expression as a prognostic marker in pancreatic cancer: a systematic review and meta-analysis

**DOI:** 10.3389/fmed.2026.1824707

**Published:** 2026-03-20

**Authors:** Oana-Iulia Cretu, Cristian Virgil Lungulescu, Manuel Gentiluomo, Adina Turcu-Stiolica, Nikola Panić, Stefan Patrascu, Bogdan Silviu Ungureanu

**Affiliations:** 1 Department of Pathology, Faculty of Medicine, University of Medicine and Pharmacy of Craiova, Craiova, Romania; 2 Department of Oncology, Faculty of Medicine, University of Medicine and Pharmacy of Craiova, Craiova, Romania; 3 Department of Biology, University of Pisa, Pisa, Italy; 4 Department of Biostatistics, Faculty of Pharmacy, University of Medicine and Pharmacy of Craiova, Craiova, Romania; 5 Department of Health Economics and Outcomes Research, Faculty of Medicine, University of Medicine and Pharmacy “Iuliu Hatieganu”, Cluj-Napoca, Romania; 6 Digestive Endoscopy Unit, University Hospital Center Dr. Dragiša Mišović Dedinje, Belgrade, Serbia; 7 Department of General Surgery, Faculty of Medicine, University of Medicine and Pharmacy of Craiova, Craiova, Romania; 8 Department of Gastroenterology, Faculty of Medicine, University of Medicine and Pharmacy of Craiova, Craiova, Romania

**Keywords:** meta-analysis, metastasis, overall survival, pancreatic cancer, vimentin

In the published article, an **Acknowledgments** section was omitted. The section should be added as follows:

## Acknowledgments

This research was partially funded by the Romanian Executive Unit for Financing Higher Education, Research, Development and Innovation, UEFISCDI Agency, Project PN-IV-P2-2.1-TE-2023-1752 No 101TE of 03/01/2025, DYOPAC.

The original article has been updated.

