# Supplementary material for: Vimentin expression as a prognostic marker in pancreatic cancer: a systematic review and meta-analysis
Source: Front Med (Lausanne). 2026 Feb 13;13:1742644. doi: 10.3389/fmed.2026.1742644 (PMC12945835; doi:10.3389/fmed.2026.1742644)
Supplement: Supplementary file 2 [file Table_2.pdf]

Table S2. The search strategy

**PubMed**	((Vimentin[MeSH Terms] OR Vimentin[Title/Abstract]) AND ("Pancreatic Neoplasms"[MeSH Terms] OR "Pancreatic Ductal Adenocarcinoma"[Title/Abstract] OR "Pancreatic Cancer"[Title/Abstract] OR "Pancreas Cancer"[Title/Abstract]) AND (Prognosis[MeSH Terms] OR Survival[Title/Abstract] OR "Overall Survival"[Title/Abstract] OR Metastasis[Title/Abstract] OR "Lymph Node"[Title/Abstract] OR "TNM stage"[Title/Abstract] OR "Clinical Stage"[Title/Abstract] OR "Histological Grade"[Title/Abstract]))
**Web of Science (Core Collection)**	TS=(Vimentin) AND TS=("Pancreatic Cancer" OR "Pancreatic Neoplasm" OR "PDAC" OR "Pancreatic Ductal Adenocarcinoma") AND TS=(Prognosis OR Survival OR "Overall Survival" OR "Lymph Node" OR Metastasis OR Stage OR Grade)
**Scopus**	TITLE-ABS-KEY (vimentin) AND TITLE-ABS-KEY ("pancreatic cancer" OR "pancreatic ductal adenocarcinoma" OR "PDAC") AND TITLE-ABS-KEY (prognosis OR survival OR "overall survival" OR metastasis OR "lymph node" OR stage OR grade)

Table S3. NOS domains per study

Study	Selection				Comparability	Outcome			Score
	1	2	3	4	5	6	7	8	
Chouat 2017	★	★	★	★	★★	★	★	★	9
Guo 2014	★	★	★			★			4
Javle 2007	★	★	★	★	★	★	★		7
Kokumai 2023	★	★	★	★	★★	★	★		8
Lee 2014	★	★	★	★	★★	★	★		8
Maehira 2019	★	★	★	★	★★	★	★	★	9
Sanchez Ramirez 2022	★	★	★	★	★★	★	★	★	9
Wang 2019	★	★	★	★	★★	★	★		8
Xu 2013	★	★	★	★	★	★	★		7

NOS, Newcastle-Ottawa Scale; 1=representativenesss of the exposed cohor, 2=selection of the non-exposed cohort, 3 = ascertainment of exposure, 4 = outcome of interest not present at the start of the study, 5 = outcome of interest not present at the start of study, 6 = assessment of outcome, 7=assessment of outcome, 8 = adequacy of follow up of cohorts.

Table S4. Technical diagnostics and outlier analysis for meta-analyses

Analysis (Outcome)	Model	Q-test (p)	Tau^2	Influence Diagnostics (Cook's Distance / Outliers)	Funnel Plot Tests (Egger's p/ Begg's p)
Overall Survival	Random	< 0.001	2.36	No influential studies or outliers detected.	0.026*<br>0.719
N-stage	Fixed	0.418	0.00	No influential studies or outliers detected.	0.09<br>0.48
M-stage	Fixed	0.324	0.00	No influential studies or outliers detected.	0.37<br>0.33
T-stage	Fixed	0.408	0.00	No influential studies or outliers detected.	0.410<br>1.0
Hist. Grade	Random	0.031	1.91	Maehira et al. showed high studentized residual.<br>No influential studies detected.	0.009*<br>0.333
Hist. Stage	Random	0.004	1.48	Sanchez Ramirez et al. identified as outlier.<br>No influential studies detected.	0.64<br>0.75

*, Significant.
